# What Do Contrast Threshold Equivalent Noise Studies Actually Measure? Noise vs. Nonlinearity in Different Masking Paradigms

**DOI:** 10.1371/journal.pone.0150942

**Published:** 2016-03-08

**Authors:** Alex S. Baldwin, Daniel H. Baker, Robert F. Hess

**Affiliations:** 1 McGill Vision Research, Department of Ophthalmology, McGill University, Montreal, Canada; 2 Department of Psychology, University of York, York, England; The Ohio State University, Center for Cognitive and Brain Sciences, Center for Cognitive and Behavioral Brain Imaging, UNITED STATES

## Abstract

The internal noise present in a linear system can be quantified by the equivalent noise method. By measuring the effect that applying external noise to the system’s input has on its output one can estimate the variance of this internal noise. By applying this simple “linear amplifier” model to the human visual system, one can entirely explain an observer’s detection performance by a combination of the internal noise variance and their efficiency relative to an ideal observer. Studies using this method rely on two crucial factors: firstly that the external noise in their stimuli behaves like the visual system’s internal noise in the dimension of interest, and secondly that the assumptions underlying their model are correct (e.g. linearity). Here we explore the effects of these two factors while applying the equivalent noise method to investigate the contrast sensitivity function (CSF). We compare the results at 0.5 and 6 c/deg from the equivalent noise method against those we would expect based on pedestal masking data collected from the same observers. We find that the loss of sensitivity with increasing spatial frequency results from changes in the saturation constant of the gain control nonlinearity, and that this only masquerades as a change in internal noise under the equivalent noise method. Part of the effect we find can be attributed to the optical transfer function of the eye. The remainder can be explained by either changes in effective input gain, divisive suppression, or a combination of the two. Given these effects the efficiency of our observers approaches the ideal level. We show the importance of considering these factors in equivalent noise studies.

## Introduction

### The equivalent noise method

The neural signals in the human visual system are perturbed by internal noise from a variety of sources [1]. According to signal detection theory [2] (SDT) this noise limits our ability to detect weak signals [3]. The detectability of a stimulus is therefore expressed as the signal-to-noise ratio *d*′. Under this analysis many psychophysical paradigms in which “thresholds” are measured can instead be thought of in terms of what is happening to the underlying noisy response distributions in, for example, the target and null intervals of a forced-choice task. In fact under SDT there is no special “threshold” stimulus magnitude, and references made to thresholds are a matter of convenience [4, 5]. The fundamental importance of understanding the visual system’s internal noise has resulted in many studies that have explicitly attempted to characterise it, and measure how it changes under various conditions [6–12].

The equivalent noise method can be used to measure the internal noise of an observer. The method is borrowed from the field of electrical engineering [13] and involves increasing the variance of external noise added to the stimulus until it causes a change in behaviour (i.e. elevating detection thresholds). At this point it is said to have exceeded the equivalent internal noise. This effect is most easily understood by considering the Linear Amplifier Model (LAM). In this model the observer receives a response on a trial-by-trial basis from the target interval

$$\begin{array}{c} r_{\text{targ}}=c+N_{\text{int}}+N_{\text{ext}}, \end{array}$$ (1)

and the null (target absent) interval

$$\begin{array}{c} r_{\text{null}}=N_{\text{int}}+N_{\text{ext}}, \end{array}$$ (2)

where *c* is the target contrast and each of the random variables $N_{\text{int}}∼N\left(\mu ,\frac{1}{2}{\hat{\sigma }}_{\text{int}}^{2}\right)$ and $N_{\text{ext}}∼N\left(\mu ,\frac{1}{2}\sigma_{\text{ext}}^{2}\right)$ are independent samples drawn from a pair of normal distributions corresponding to the internal and external noise. These are defined using the notation $N(\mu ,\sigma^{2})$ to give their mean *μ* and variance *σ*^2^. The observer makes their decision by taking the difference between the responses from the two intervals

$$\begin{array}{c} \Delta r=c+N_{\text{int}}+N_{\text{ext}}-N_{\text{int}}^{\prime }-N_{\text{ext}}^{\prime }. \end{array}$$ (3)

Note that the noise samples from the null interval are distinguished from those from the target interval by the addition of prime symbols. This is to indicate that there are two independent random variables sampled from both the internal and external noise sources (one sample in each interval). These will have different values (and so do not simply cancel) but the same underlying statistics. Therefore this equation simplifies to

$$\begin{array}{c} \Delta r=c+X_{\text{int}}+X_{\text{ext}}, \end{array}$$ (4)

where $X_{\text{int}}∼N\left(0,{\hat{\sigma }}_{\text{int}}^{2}\right)$ and $X_{\text{ext}}∼N\left(0,\sigma_{\text{ext}}^{2}\right)$. The signal-to-noise ratio (*d*′) is

$$\begin{array}{c} d^{\prime }=\frac{c}{\sqrt{{\hat{\sigma }}_{\text{int}}^{2}+\sigma_{\text{ext}}^{2}}}, \end{array}$$ (5)

and solving for *d*′ = 1 gives the threshold contrast at *P*_correct_ = 76.02%

$$\begin{array}{c} c_{\text{threshold}}=\sqrt{{\hat{\sigma }}_{\text{int}}^{2}+\sigma_{\text{ext}}^{2}}. \end{array}$$ (6)

One can see in Eq (6) that thresholds will largely be determined by the dominant noise source. When ${\hat{\sigma }}_{\text{int}}\gg \sigma_{\text{ext}}$ the threshold will be approximately ${\hat{\sigma }}_{\text{int}}$ and when ${\hat{\sigma }}_{\text{int}}⪡\sigma_{\text{ext}}$ the threshold will be approximately *σ*_ext_. Where the *σ*_ext_ value is given in either the same or at least equivalent units to *c*, this is the behaviour of an ideal observer with internal noise. Empirically however one very often finds that thresholds in dominant external noise are higher than is predicted by this model. This cannot be due to the internal noise, as it is already established that its effects are negligible when the external noise is dominant. These effects are instead accounted for using a “calculation efficiency” parameter, *η*, so that

$$\begin{array}{c} c_{\text{threshold}}=\frac{\sqrt{{\hat{\sigma }}_{\text{int}}^{2}+\sigma_{\text{ext}}^{2}}}{\eta }. \end{array}$$ (7)

Where the *σ* values are given in either the same or at least equivalent units to *c*, this is the efficiency relative to an ideal observer with internal noise (ideal *η* is 1). Otherwise the value of *η* only has a relative meaning (as the ideal *η* is unknown). One factor that can affect efficiency is the nature of the integration stage (see Appendix B).

Eq (7) is frequently used as a model of how thresholds are expected to change with different levels of external noise (*σ*_ext_), and then to infer both the internal noise (${\hat{\sigma }}_{\text{int}}$) and the efficiency (*η*). If human vision behaves like the LAM (linear signal transduction and additive noise) then differences in performance can be wholly explained by these two parameters. This has been used to investigate contrast detection in normal observers [13, 14], in disease [15–17], and in ageing [18–21]. However, there are several objections to this approach, as we now outline.

### First objection: nonlinearities

Studies that use the LAM to fit contrast threshold data and then report ${\hat{\sigma }}_{\text{int}}$ as the noise in the brain assume that the transduction of contrast is linear and that the noise is additive. The first assumption is challenged by multiple studies that have provided evidence of nonlinear transduction [22–26]. The nonlinearity is typically described as accelerating at low contrasts and saturating at high contrasts. A model of this form was provided by Legge & Foley in 1980 [22]. This model was later modified [27] to introduce a saturation constant which represents a general suppression from divisive gain control processes. In this model the contrast response function transforms the stimulus contrast

$$\begin{array}{c} \text{f}\left(c\right)=\frac{c^{p}}{z+c^{q}}, \end{array}$$ (8)

where the exponents *p* and *q*, and the saturation constant *z*, control the shape of the function. Considering a pedestal masking experiment on a trial-by-trial basis, the observer receives a response from the target interval

$$\begin{array}{c} r_{\text{targ}}=\text{f}\left(t+m\right)+N_{\text{int}}, \end{array}$$ (9)

and the null interval

$$\begin{array}{c} r_{\text{null}}=\text{f}\left(m\right)+N_{\text{int}}, \end{array}$$ (10)

where *t* and *m* are the target and mask contrasts respectively. Therefore the *d*′ for an observer discriminating between mask (*m*) and target plus mask (*t* + *m*) intervals is

$$\begin{array}{c} d^{\prime }=\frac{f(t+m)-f(m)}{\sigma_{\text{int}}}. \end{array}$$ (11)

Note that the *σ*_int_ on the denominator represents the noise across both intervals of the 2IFC task, as is also the case in Eq (7).

Models of this general form have been shown to account for behaviour in psychophysical experiments [22, 27–30], as well as the fMRI BOLD response [25], and electrophysiological responses from steady-state EEG [31] and single-cell activity [32]. A model featuring this nonlinearity has been suggested to explain results from noise masking studies [33, 34], and it has been shown previously that the stochastic resonance effect (where low levels of noise *facilitate* detection) can be explained by a model of this type [35]. Once the linearity assumption is violated the change in ${\hat{\sigma }}_{\text{int}}$ does not necessarily indicate a change in internal noise, and may instead be due to other factors. For this reason we use *σ*_int_ to refer to the internal noise in our nonlinear model (Eq 11) and ${\hat{\sigma }}_{\text{int}}$ to refer to the internal noise as determined by the LAM (Eq 7) Although this caveat is recognised by leading proponents of noise masking [11] it is often overlooked when the paradigm is applied, particularly in clinical studies (e.g. [17, 21]).

**Fig 1:**
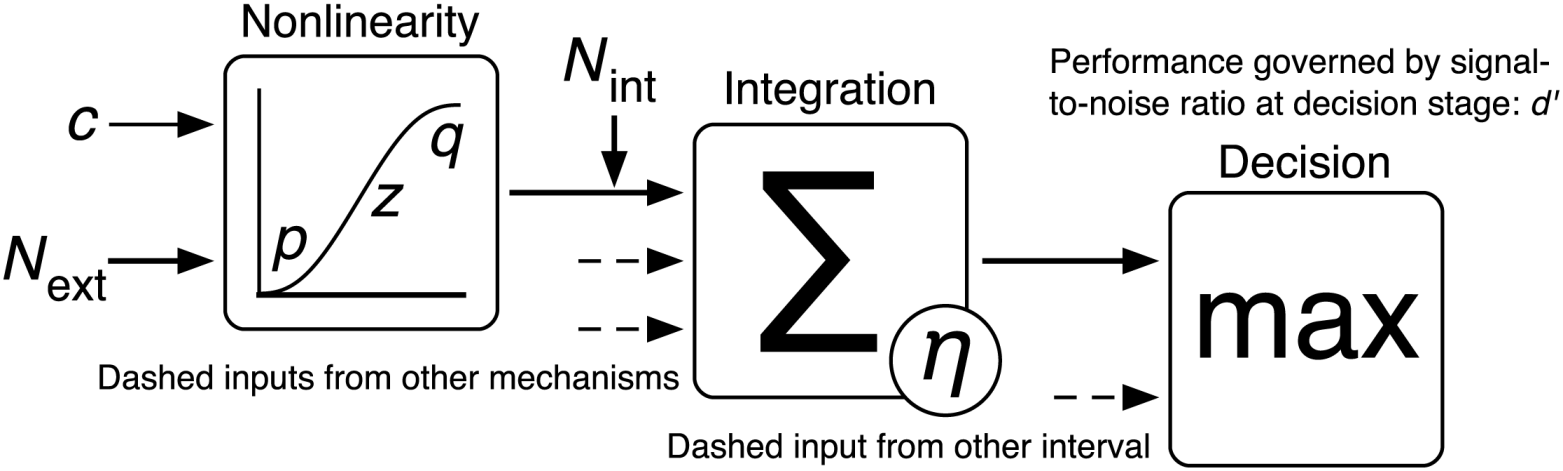
Diagram of an example model showing how contrast may be processed by the visual system. The pathway for a single mechanism in a single interval is shown in full. Other mechanisms and intervals are implied by the dashed arrows. The tuned response to the target (*c*) and any external noise falling within the mechanism’s passband (*N*_ext_) undergoes a nonlinear transformation (the mutual suppression pathways between the mechanisms in this stage have been omitted for clarity). Each mechanism is then affected by internal noise (*N*_int_) and then some integration over their outputs is performed (with its behaviour resulting in a characteristic efficiency *η*). The observer then makes a decision based on which of the two intervals has the greater response.

A diagram of the model we put forward in this paper is provided in Fig 1 and described in the figure caption. To be clear: when we refer to response levels (*r*) these are abstractions of the level of activity (e.g. neural firing rate) that is being input into the decision stage. When we talk about *d*′ we are referring the signal-to-noise ratio at the decision stage (that determines the discriminability of the target and null intervals). Note that the integration stage plays no direct role in the modelling of our experiments (though it is discussed in Appendix B), as our stimuli were relatively small and we did not manipulate their spatial extent. It is included in the diagram for the sake of consistency with other established results [36–38]. An alternative to a model featuring a nonlinear contrast response would be one where the noise scales multiplicatively with the signal. The most popular of these is the perceptual template model (PTM) [10]. This is a popular model which has supplanted the LAM in some areas [39–41]. It addresses the two principal failings of the LAM, which i) predicts greater double pass consistency than is empirically observed [42], and ii) predicts psychometric functions that are shallower than those found experimentally. The PTM fixes these problems by adding a nonlinear transducer and a late-acting multiplicative noise term that is proportional to the mask contrast. It has been shown however that the discrepancies between the LAM predictions and the data can be explained by gain control effects arising from the broadband masking noise typically used (see below [43, 44]). Furthermore, the induced multiplicative noise the PTM relies on may not be justified. An experiment featuring simultaneous cross-channel and noise masking has shown behaviour inconsistent with the induced multiplicative noise in the PTM, favouring instead a divisive gain control model [44]. Another study that measured the ratio between the internal noise standard deviations at different contrast levels found that it did not increase with contrast, and that the masking was due to a compressive nonlinearity [45]. For further discussion of the limitations of the PTM see also Goris et al. [30].

### Second objection: the masking noise

Most noise masking experiments that look at contrast detection use broadband noise, however this will have an additional effects from cross-channel masking that are not incorporated into standard models [43, 44]. Most of the energy in a broadband mask will fall outside the passband of the mechanism that is being investigated. Mechanisms with different tunings will detect these other spectral components, and their activation will result in divisive suppression of the target mechanism through the gain control pool [24, 26, 27, 44, 46]. As most of the noise masking literature uses broadband noise [47, 48] it is difficult to distinguish the effects which can be attributed to noise masking from those which may result from cross-channel masking. These problems can be avoided by using “zero-dimensional” contrast-jitter noise [43]. In this method the noise used is a version of the target stimulus with different randomised contrasts added to both the target and null intervals (see also Cohn’s work [49] on the detection of luminance increments). It is superior in that it performs the exact role specified for the external noise in the equivalent noise method while minimising the additional effects introduced by other types of noise.

Previous experiments using contrast-jitter noise have shown that the principal failings of the LAM (listed above) that are used to justify the PTM are instead due to the use of broadband noise [43, 44]. If zero-dimensional noise is used then the expectations of the LAM are met (double pass consistency is higher and the psychometric function is shallower). The contrast-jitter noise is analogous to the way in which equivalent noise experiments are typically performed in other domains such as orientation [50]. By switching to this type of noise and removing the cross-channel effects (which are rarely considered in previous studies) we end up with a task that more clearly distinguishes the pedestal masking and noise masking paradigms [44]. It is on this basis that we reconsider the relationship between the information available from those two tasks here.

### This study

In this study we compare pedestal and contrast-jitter noise masking functions using the same stimuli within a set of observers in order to demonstrate that noise masking results that have previously been attributed to internal noise effects may be entirely due to changes in gain control. We chose to make the comparison between two spatial frequencies (0.5 and 6 c/deg) as the loss of sensitivity with increasing spatial frequency is well-established by the many experiments that have measured the contrast sensitivity function (CSF). Although some of this effect can be attributed to the eye’s optics, it is clear that neural factors also play a role [51]. The LAM fits made in Fig 2.1 of Pelli’s thesis on detection in noise [13] indicate the change in sensitivity with spatial frequency is due to both an increase in internal noise coupled with a loss of efficiency. More recent studies have sought to use the noise masking method to explain how the CSF changes with age [18–20, 52]. We will address the various factors that may be responsible for the shape of the CSF in our discussion section.

## Methods

### Observers

Five psychophysically-experienced observers took part, including two authors (AB and DB). All observers were tested at McGill University except DB, who was tested at the University of York. Observers either had normal vision, or wore their prescribed optical correction appropriate to the viewing distance. The experiment was carried out with informed written consent in accordance with tenets of the Declaration of Helsinki, and approved by the Centre for Applied Ethics at the McGill University Health Centre.

### Equipment

All experiments were conducted using Matlab 2010a. A Cambridge Research Systems Visage was used to provide 14 bits of contrast depth on a gamma-corrected CRT monitor. At McGill the stimuli were presented on a Dell Trinitron with a mean luminance of 83 cd/m^2^. These experiments were conducted with natural pupils, however measurements of pupil diameter under the testing conditions were made for each observer. These were within the range of 2.5-3.5 mm. At York the monitor was an Iiyama VisionMaster Pro 510, and the mean luminance was 37 cd/m^2^. DB had a pupil diameter of approximately 5.5-6 mm.

### Stimuli

**Fig 2:**
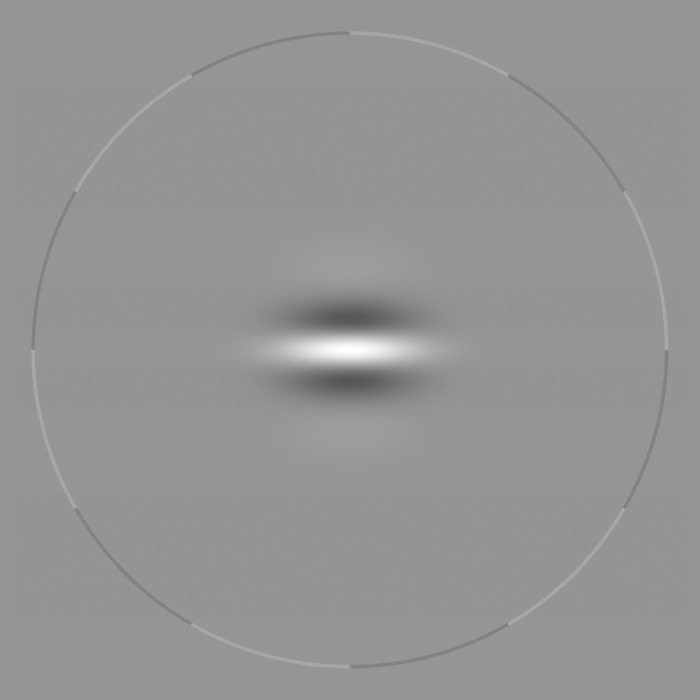
An example of the log-Gabor stimuli used in these experiments. The stimulus is shown within the fixation ring used to reduce uncertainty.

We used cosine-phase horizontal Cartesian-separable log-Gabor [53, 54] patches(Fig 2). These are defined in the Fourier domain as log-Gaussians radially (controlling the frequency bandwidth) multiplied by orthogonal Gaussians (controlling the orientation bandwidth). They had spatial frequency bandwidths of 1.6 octaves and orientation bandwidths of ±25°, giving them a similar profile to that of a simple cell receptive field [55, 56]. Their envelopes have a full width at half magnitude of 0.91 cycles vertically, and 1.17 cycles horizontally. An advantage of using log-Gabor stimuli is that they are DC-balanced (do not affect global mean luminance) in any phase. We report their delta-contrast calculated from a luminance matrix **L** with mean luminance *L*_mean_

$$\begin{array}{c} c_{\text{delta}}=\frac{\text{max}(|\text{L}-L_{\text{mean}}|)}{L_{\text{mean}}}. \end{array}$$ (12)

These contrasts are reported in dB *c*_dB_ = 20 × log_10_(*c*_delta_). For convenience we also express some of our model parameters in this same unit. Two spatial frequencies were used in both experiments: 0.5 c/deg and 6 c/deg. Our viewing distance was set to give 48 pixels per degree of visual angle, so the numbers of pixels per cycle were 96 and 8 respectively. The viewing distance at both testing locations was 80 cm.

### Procedures

All experiments were conducted using a two-interval forced-choice procedure. The stimulus duration was 100 ms, with both inter-stimulus and inter-trial (post-response) delays of 400 ms. Target contrast was controlled by a pair of staircases. One was three-down one-up and the other two-down one-up, so that the combined data from the two would concentrate sampling at the 70.71% and 79.37% points on the psychometric function (helping us obtain good estimates of psychometric slope). Although the staircases may not actually converge to these points [57] this is not important, as we obtain our threshold estimates by fitting Weibull psychometric functions in *Palamedes* [58] and then using the inverse of the Weibull to find the threshold level that corresponds to 76.02% (where *d*′ = 1).

The other properties of the staircases were a 6 dB step size before the first reversal followed by a 3 dB step size thereafter, and a stopping point of 70 trials or 12 reversals (whichever was reached first). In the noise masking experiment each condition was repeated four times. In the pedestal masking experiment there were three repetitions. In both cases the data were collapsed across repetition and psychometric function fits performed on the combined data. Appendix A includes an example psychometric function from the unmasked condition fitted by both a Weibull function and the CRF model we introduce later.

Observers were only tested on one spatial frequency in each block. To reduce spatial uncertainty and provide a continuous fixation cue the stimuli were surrounded by a ring (radius of 3.75 carrier cycles), as seen in Fig 2. The ring inverted from light to dark around its circumference (with a contrast of 15%) meaning it had no effect on the mean luminance. To reduce temporal uncertainty the contrast of the ring inverted during stimulus presentation, and a short beep was played at stimulus onset. Observers responded as to which interval contained the higher contrast with the buttons of a mouse, following which they were given feedback (high- or low-pitched beeps) based on whether they were correct.

For the pedestal masking experiments a version of the target with an identical pedestal contrast was added to both intervals in each trial. This pedestal contrast was kept constant in each testing block. The mask levels were chosen based on the observer’s detection thresholds, as the “dip” in these functions typically occurs when the pedestal contrast is equal to the unmasked threshold. Our general method was to test at -12, -6, -3, 0, +3, +6, +12, and +18 dB relative to the threshold measured for each spatial frequency, and also fixed points at the minimum of 0% contrast (no mask) and maximum of 44.7% (33 dB). In practise this resulted in some observers for whom mask levels would either overlap or fall out of range, so for these they were altered slightly.

For the noise masking experiments we used the “zero-dimensional” contrast-jitter noise [43], where an independently-generated random contrast offset is added to each interval. These are drawn from a normal distribution with its mean at zero and its standard deviation determining the masking noise level. We collected data both without a mask (0%, in addition to the identical condition tested in the pedestal masking experiments), and with noise standard deviations of 18, 21, and 24 dB. Trials where the contrast of the jitter noise was greater than 55% had the contrast reduced to that value (this affected at most 0.05% of trials in the 24 dB condition). Crucially, the stimulus contrasts in this method can be “negative”, in which case the stimulus polarity is reversed. For this reason we instruct our observers to select the interval with the highest “positive” contrast, where it is bright in the centre. In the noise masking experiments it is possible for the noise to cause the stimulus in the null interval to be a better “target” (higher positive contrast) than the one in the target interval. This is essentially true of all noise-masking experiments and is the reason why the ideal observer prediction is that thresholds should increase in proportion to the standard deviation of the masking noise. For the purpose of providing feedback to our participants we give the “correct” beep whenever they pick the interval with the higher positive contrast, whereas for data collection purposes we still classify correct and incorrect responses based on which interval was initially defined as the target before the noise was added.

It is important to recognise that the two paradigms we use are closely related. In both pedestal and noise masking one can consider the observer’s task to consist of discriminations made in a two-dimensional space where the *x*-axis is the response to the null interval and the *y*-axis the response to the target interval. In a pedestal masking experiment the expected *x* and *y* coordinates on each trial are specified by the target and pedestal contrasts. In a noise masking experiment the contrasts in each interval are chosen randomly (albeit with specific means and standard deviations). The actual contrasts presented in each interval are not usually used in the analysis of a noise masking experiment (instead the mean and standard deviation are used), however if one were to save this information it would be possible to rearrange data from sufficiently extensive testing in a way that it could be analysed like the data from a pedestal masking experiment. The reverse is also true: by including a wide range of target and pedestal contrasts it would be possible to collect a pedestal masking data set whose trials could be rearranged into those that would be collected in a noise masking experiment. Therefore, any explanation of the results from one experiment should be consistent with the results from the other.

### Bootstrapping

When fitting our psychometric functions, we used the parametric bootstrapping routines in *Palamedes* to obtain a population of 1,000 bootstrap samples from which we could calculate a median and 95% confidence interval. We use the median as the central tendency when we plot our data because it is the measure most consistent with the use of the 95% confidence interval from bootstrapping to represent the variability associated with that value. In reality however the use of the median from bootstrapping rather than the single threshold obtained by fitting to the raw data made very little difference. Combining data across all conditions and observers and then subtracting the raw thresholds for each psychometric function from the medians obtained by bootstrapping gives us a distribution of residuals with a mean of 0.01 dB, a standard deviation of 0.05 dB, and a maximum difference of 0.25 dB.

We also used bootstrapping to obtain confidence intervals and standard errors for our model-fitting. For the likelihood-based modelling we performed for our pedestal masking experiments we randomly sampled individual trials (with replacement) from our empirical data to obtain 1,000 bootstrap datasets. Parameter bootstrap distributions were obtained by fitting the model to these datasets. For the noise masking experiments we obtained parameter bootstrap distributions by fitting to the threshold populations generated by *Palamedes*. For our figures, we simply present the medians and 95% confidence intervals of these distributions. When calculating standard error (the standard deviation of the population of bootstrap samples) for our tables however, the effects of a few outlier bootstrap samples would occasionally exaggerate the standard errors when compared to the confidence intervals. These outliers occur because the bootstrapping procedure will sometimes generate sample datasets that do not adequately constrain the model fit.

To prevent outliers inflating the standard error values, we employed an outlier-removal method based on that proposed by Tukey [59, 60]. We calculated the interquartile range of our bootstrap distribution IQR = Q3 − Q1, and then removed any values below Q1 − 6 × IQR or above Q3 + 6 × IQR. We chose to use a more conservative method than Tukey’s (who proposed 1.5 × IQR) in order to ensure that the outliers we removed were very far from the “true” bootstrap distributions. Removing outlier values had no effect on the standard errors reported for our pedestal masking experiment (the only outliers removed were two out of the thousand bootstrapped *σ*_int_ samples in the 6 c/deg condition for observer AR). In the LAM fitting for the noise masking experiment outliers were slightly more common. The most affected case was the 6 c/deg condition for observer AR where we removed nine out of the thousand bootstrapped *η* samples. In the fits to the simulated data used to generate the nonlinear model (NLM) prediction, it was only in that same condition for the same observer that outliers were found. Three of the thousand bootstrap samples were rejected for both the ${\hat{\sigma }}_{\text{int}}$ and *η* parameters.

## Results and Modelling

### Experiment 1: pedestal masking

**Fig 3:**
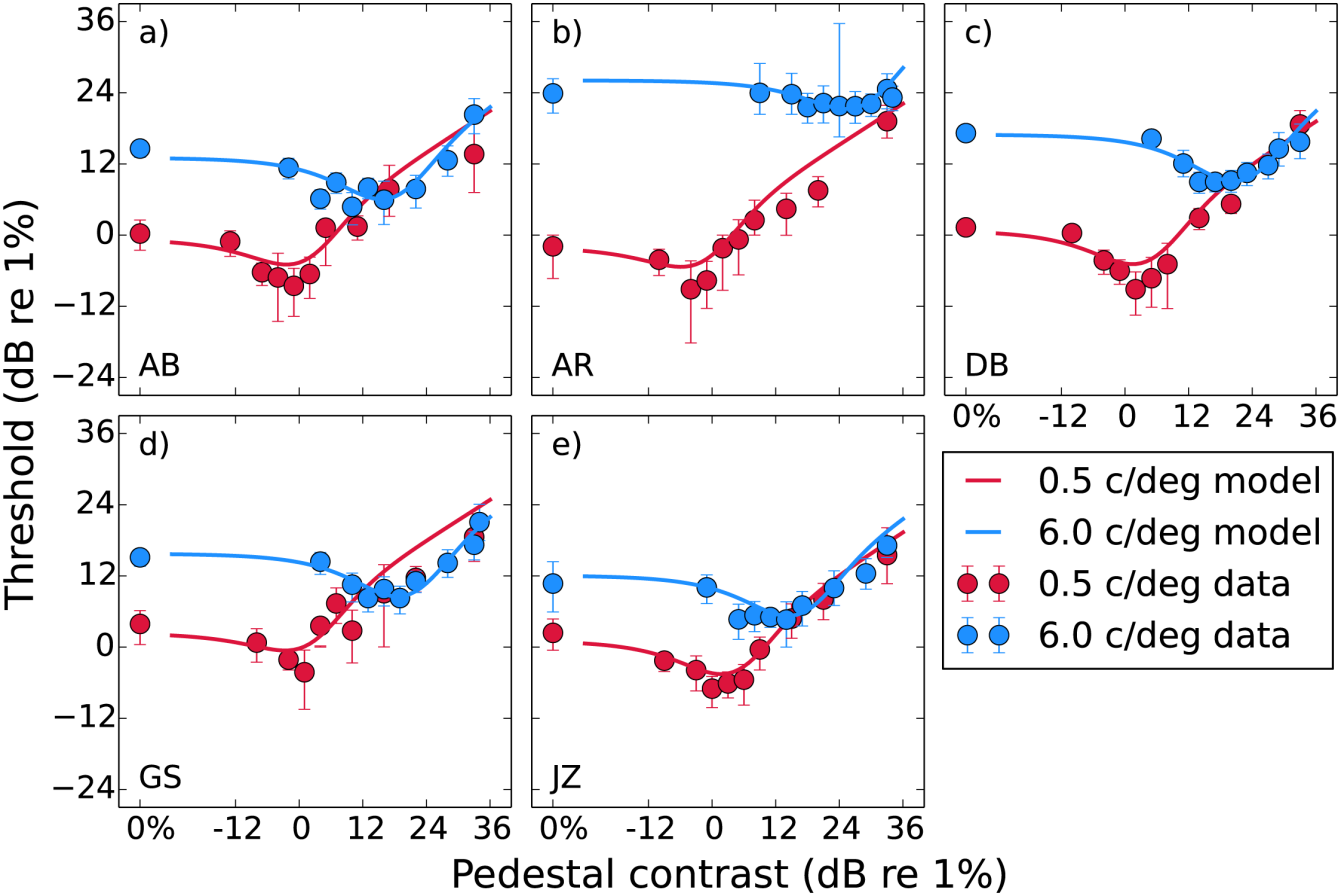
Pedestal masking (“dipper”) functions for our five observers. Median thresholds obtained from bootstrapping are plotted against pedestal mask contrast, with error bars indicating the 95% confidence intervals. The curves show predictions from Eq (11) fitted to the raw data. The parameters for these fits are provided in Table 2.

Pedestal masking functions for each of the five observers are shown in Fig 3. Each panel shows a different observer, with the two spatial frequencies shown in different colours. The thresholds have the typical “dipper” shape where lower pedestal levels produce facilitation (reduced thresholds) and higher pedestal levels produce masking (elevated thresholds), though for observer AR (Fig 3b) at 6 c/deg the magnitude of the dip is quite small (see Table 1). Consistent with previous results the pedestal contrasts that provide the greatest facilitation roughly coincide with the unmasked detection thresholds at *d*′ = 1 [34, 61].

**Table 1:** Magnitudes of the dips (differences between the unmasked and lowest masked thresholds) and slopes of the handles (straight line fits to the data where the pedestal level was >3 dB above that which gave the lowest threshold) for the data shown in Fig 3. The mean across observers is reported with its standard error.

	Dip magnitude (dB)		Handle slope	
	0.5 c/deg	6 c/deg	0.5 c/deg	6 c/deg
AB	8.8	9.8	0.48	0.83
AR	7.2	2.3	0.67	0.24
DB	10.4	8.2	0.91	0.53
GS	8.1	6.9	0.52	0.74
JZ	9.4	6.1	0.74	0.69
Mean	8.8 ± 0.5	6.7 ± 1.3	0.66 ± 0.08	0.61 ± 0.10

Comparing the results between the two spatial frequencies, the dipper functions translate upwards (higher thresholds) and to the right (higher pedestal contrast required to induce a dip) as the spatial frequency is increased. For all observers except AR this results in equal thresholds for the two spatial frequencies at the highest mask levels. This effect can also be seen in data from previous studies [62]. The average dip magnitude (difference between unmasked and lowest masked threshold) and handle slope (straight line fit to the data beyond that lowest point) are reported in Table 1. We performed paired-sample t-tests in R [63] and found no significant difference in either the mean dip across observers (*t*(4) = 2.14, *p* = 0.10), or the mean handle slope (*t*(4) = 0.37, *p* = 0.73). We shall return to these data and explain the fitted curves when we discuss the modelling.

**Fig 4:**
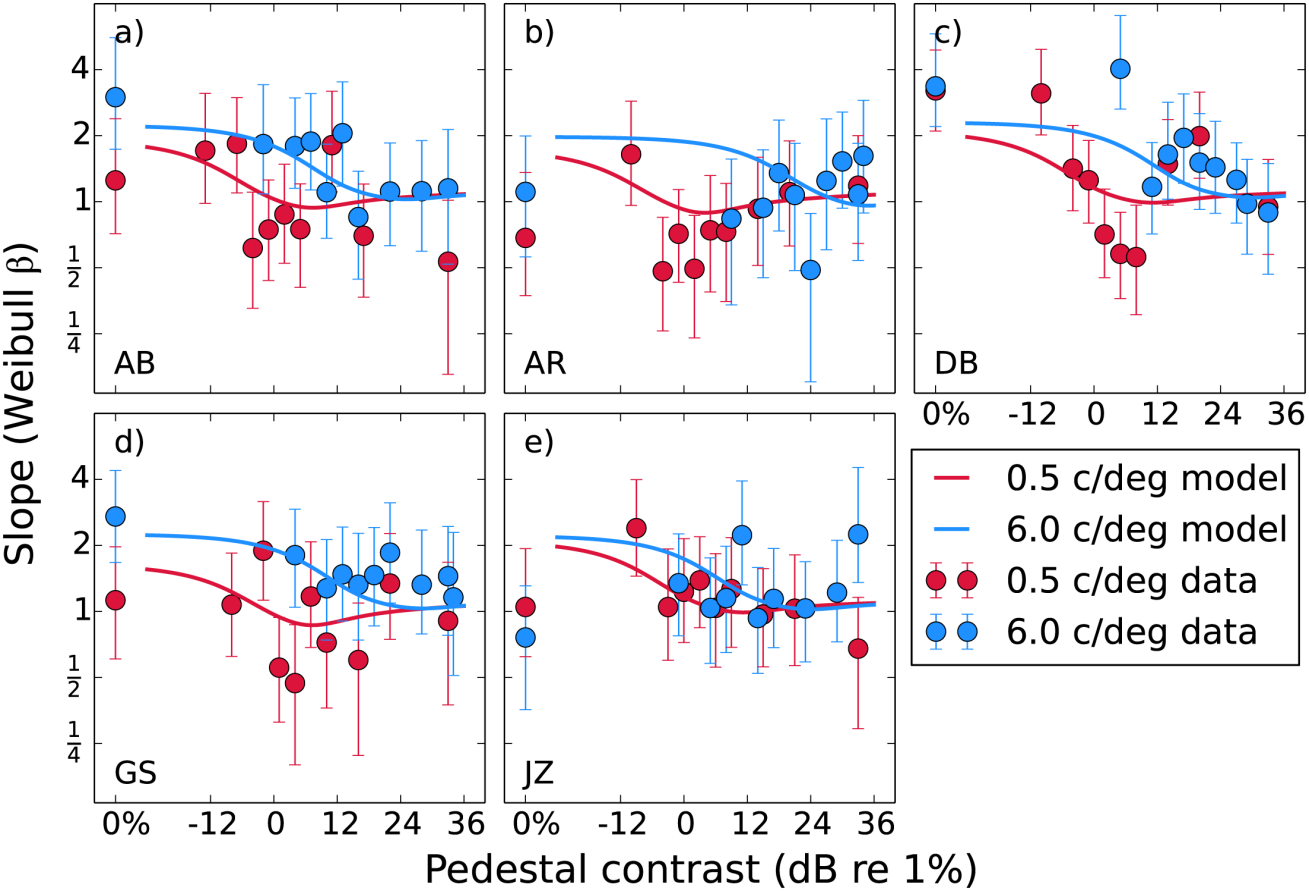
Median psychometric slopes obtained from bootstrapping plotted as a function of pedestal mask contrast for each observer. The error bars show 95% confidence intervals. As in Fig 3, the curves show the predictions from Eq (11).

The psychometric function slopes (Weibull *β*) are plotted in Fig 4. In line with previous results [28] unmasked slopes are typically more steep (mean across observers and spatial frequencies is 1.8) and become more shallow as the mask level increases (mean for the 33 dB point is 1.2). The point at which the most pronounced decrease in slope occurs appears to be that where we see the dip for the thresholds. This effect has been predicted previously [64] and is a feature of our model (fitted curves). We compared the slopes for the unmasked and 33 dB mask conditions with a t-test and found the difference not to be significant (*t*(9) = 1.62, *p* = 0.14), however we do not rely on these slopes to support our conclusions. We present them here merely for illustrative purposes.

We fitted our data with the nonlinear transducer model described by Eq (11). The parameters are the accelerating and saturating exponents *p* and *q*, saturation constant *z*, and internal noise *σ*_int_. Rather than fitting the model to the thresholds obtained from psychometric function fitting, as is typical, we instead implemented a maximum-likelihood method that allowed us to fit to the raw data (an explanation is provided in Appendix A). This more powerful method creates a prediction that is two-dimensional: a dipper along one dimension and a psychometric function along the other [65]. Although we do not fit to the thresholds and slopes that were obtained by Weibull fitting we can still generate model predictions for them. These are shown by the curves in Figs 3 and 4. For the dipper function an increase in the *z* parameter does not affect the “handle” region on the right where masking is dominant, but does translate the left part of the curve (from the unmasked threshold to the facilitative dip) diagonally upward and to the right. An increase in the *σ*_int_ on the other hand translates the entire curve directly upward.

**Fig 5:**
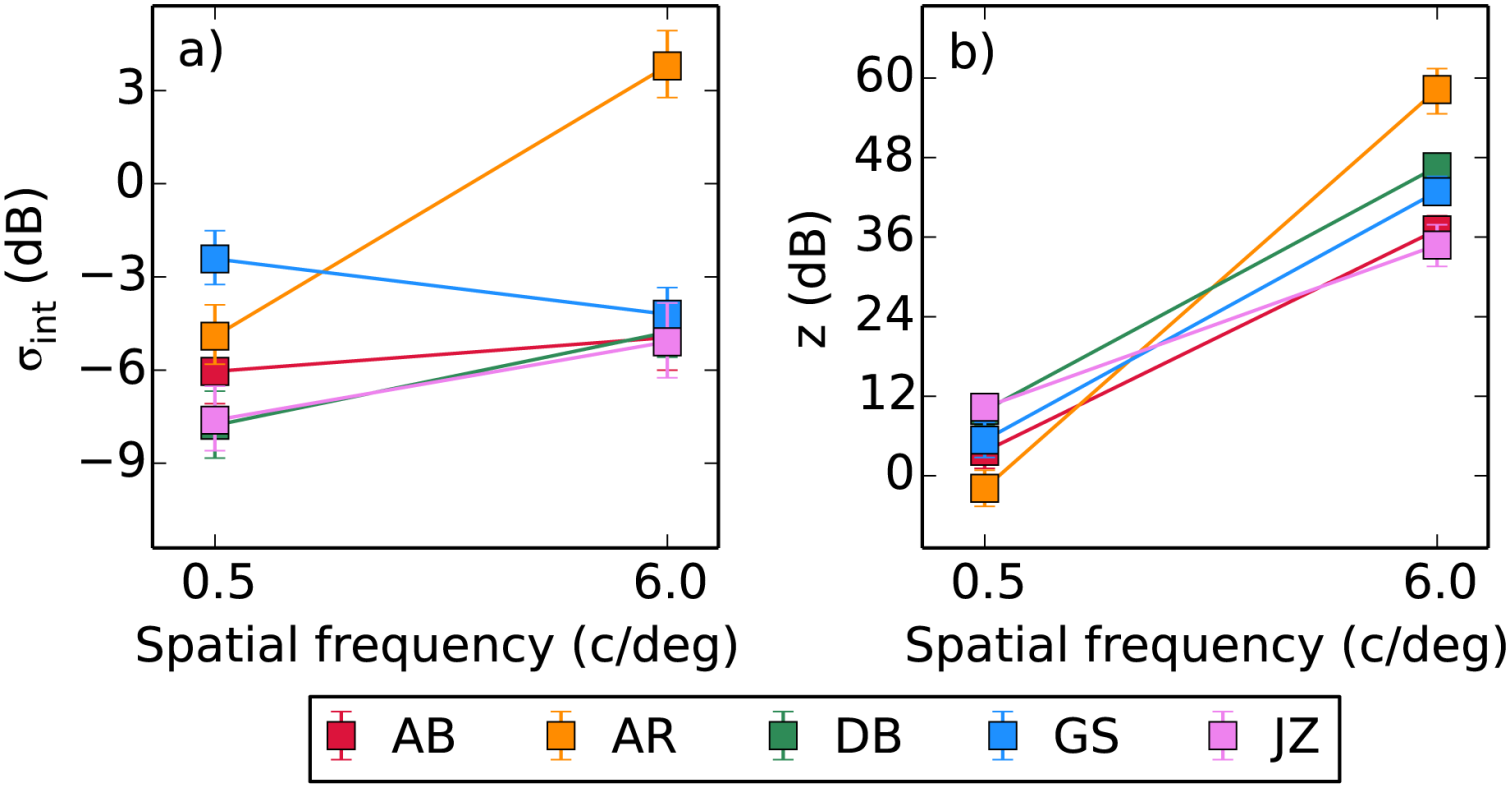
Median parameter estimates obtained by fitting to the bootstrapped pedestal masking data, with 95% confidence intervals. These values differ slightly from those in Table 2 as those were obtained by fitting to the empirical data.

Based on previous results [22] we fixed the values of the exponents *p* and *q* at 2.4 and 2 respectively. These values provide good fits to the data here, as they have also done in other studies [29, 66, 67]. We then fitted the remaining two parameters in order to find the values that maximised the likelihood of the fit, reported in Table 2. We also calculated the deviance (*D*) of the fits relative to the saturated model [68]. Immediately there appears to be a much greater difference in *z* than *σ*_int_ between the two spatial frequencies. Over the 12-fold increase in spatial frequency there was an 84-fold increase in *z*, but only a 1.4-fold increase in *σ*_int_. As the change in *σ*_int_ was small we considered a model where it was fixed across spatial frequency, however the AIC_*c*_ (corrected Akaike’s Information Criterion) scores of this model were higher and so the inclusion of individual parameters was preferred [69, 70]. We also fitted the model to each of the thousand sample datasets generated by a nonparametric bootstrapping procedure (sampling individual trial responses with replacement), and report the median fitted parameters with 95% confidence intervals in Fig 5. The changes in *z* vastly exceed the confidence intervals provided by the bootstrapping. Although the changes in *σ*_int_ are much smaller the confidence intervals only overlap for AB, indicating that the small differences present for the other observers are still significant.

**Table 2:** Fitted model parameters (in dB) for each observer, with the log-likelihoods of the fits and the deviances relative to the saturated model. Standard errors provided are calculated from the bootstrap distributions. Briefly, differences in *z* indicate changes in contrast gain whereas *σ* is the standard deviation of the internal noise. The predictions based on these parameters are shown in Figs 3 and 4. The mean across observers is reported with its standard error.

	*z* (dB)		*σ*_int_ (dB)			
	0.5 c/deg	6 c/deg	0.5 c/deg	6 c/deg	logL	*D*
AB	3.6 ± 1.3	37.1 ± 1.2	-6.0 ± 0.5	-5.0 ± 0.5	-2836	296
AR	-1.9 ± 1.4	58.3 ± 3.4	-4.9 ± 0.5	3.8 ± 0.6	-3146	344
DB	9.8 ± 1.2	46.6 ± 0.8	-7.7 ± 0.6	-4.8 ± 0.4	-3755	346
GS	5.4 ± 1.4	42.9 ± 1.0	-2.4 ± 0.4	-4.2 ± 0.4	-2952	293
JZ	10.3 ± 1.0	34.8 ± 1.6	-7.6 ± 0.6	-5.1 ± 0.6	-2917	316
Mean	5.4 ± 2.2	44.0 ± 4.2	-5.7 ± 1.0	-3.0 ± 1.7		

### Experiment 2: noise masking

**Fig 6:**
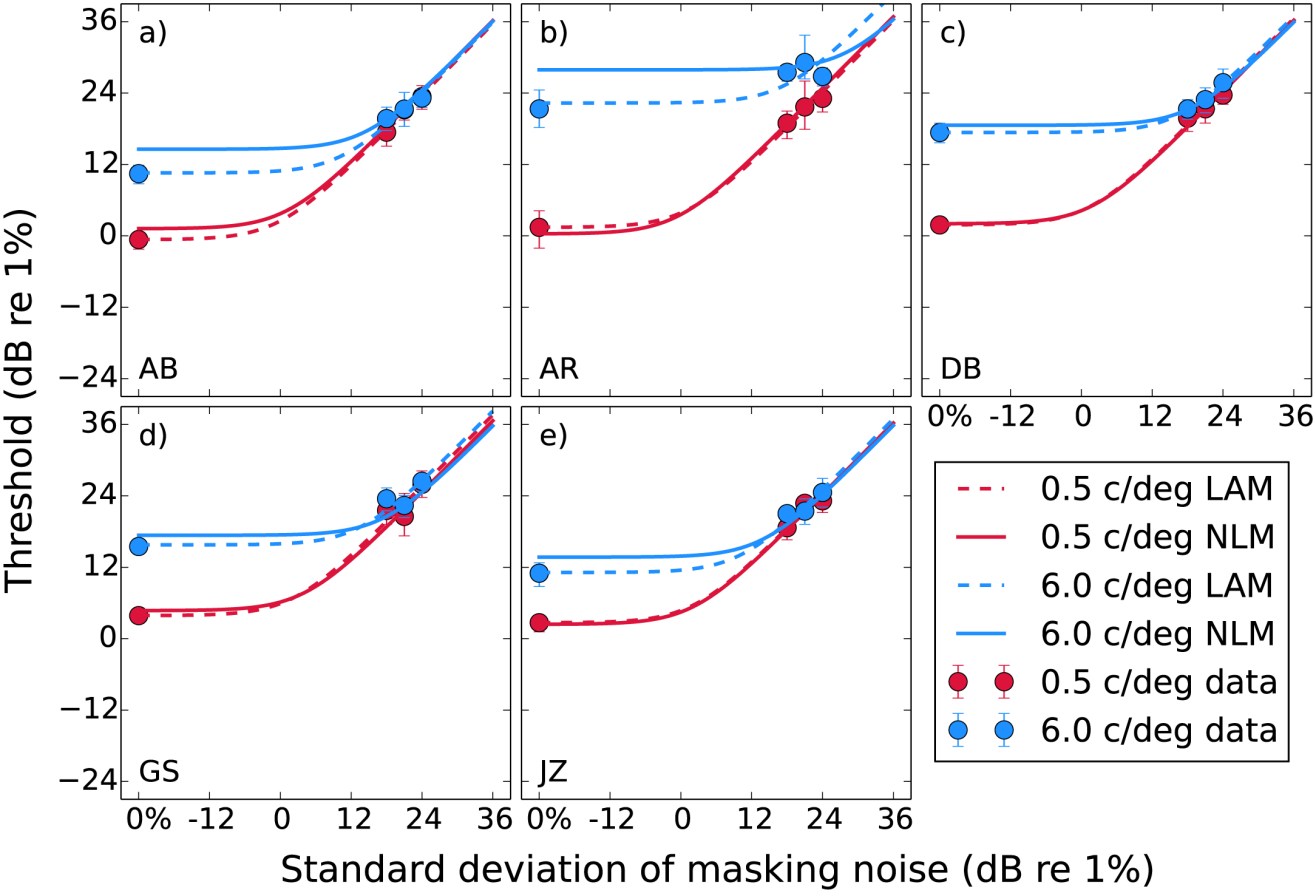
Noise masking functions for our five observers. Median thresholds obtained from bootstrapping are plotted against the standard deviation of the masking noise, with error bars indicating the 95% confidence intervals. The curves show predictions from the LAM and NLM fitted to the median thresholds. The parameters for these fits are provided in Tables 3 and 4.

In our noise masking experiments we obtain the typical result where thresholds increase with the standard deviation of the masking noise (Fig 6). The unmasked thresholds are higher for 6 c/deg than for 0.5 c/deg, however at high mask levels the results from the two spatial frequencies converge. Note that the masked thresholds roughly correspond to the standard deviation of the masking noise, which is the behaviour expected from an ideal observer. The exception is the observer AR (Fig 6b), whose noise masking curves do not converge. This was also the case for their dipper functions. It appears AR has a reduced efficiency for the 6 c/deg stimuli. Despite our efforts to control for this factor, it is possible that the the small target size is making this observer uncertain of its location (see Appendix B).

We performed a brief analysis of the psychometric slopes (Weibull *β*). Looking at the data, the mean slope decreased from 2.0 for the unmasked conditions to 1.5 for the conditions with masking noise. This is consistent with Birdsall linearisation [71] where dominant external noise shallows psychometric slopes in nonlinear systems [42, 43], though the linearisation falls short of reaching the expected slope of Weibull *β* = 1.3. A two-way within-subjects ANOVA (factors of spatial frequency and masking noise level) found no significant effects or interactions however, meaning that this decrease in slopes was not statistically significant.

The dashed curves in Fig 6 are the fitted LAM (Eq 7) functions. The parameters for these fits are provided in Table 3. The LAM provides a good fit to the data, with most of the change between the two frequencies being due to an increase in internal noise ${\hat{\sigma }}_{\text{int}}$. The solid curves in Fig 6 show predictions generated by the nonlinear model (NLM). These are not fitted to the noise masking data, but are instead calculated based on the parameters obtained from the dipper functions in Experiment 1. The contrast response function (Eq 8) is used in the equivalent noise model (Eq 3) to give

$$\begin{array}{c} \Delta r=\text{f}(c+N_{\text{ext}})-\text{f}(N_{\text{ext}}^{\prime })+X_{\text{int}}. \end{array}$$ (13)

The threshold is the value of *c* where the expected mean of a population of Δ*r* samples is equal to its expected standard deviation (i.e. *d*′ = 1). In order to approximate this value and so generate noise masking predictions, we used Eq (13) as the generating model in a Monte Carlo simulation. We generated 500 samples at 1,161 values of *c* (-18 dB to 40 dB in 0.05 dB steps) and 6 mask levels (-21 dB to 39 dB in 12 dB steps). For each mask level we found the value of *c* where the difference between the mean and standard deviation of the population of Δ*r* samples was at its minimum. This gave us six simulated data points that in all cases followed the shape expected by the LAM (initially flat, followed by a unity slope).

**Table 3:** Parameters obtained by fitting the LAM model to the data for each observer, with the RMS error of the fit. Standard errors are calculated from the bootstrap distributions. Predictions for these parameters are shown by the dashed curves in Fig 6. The mean across observers is reported with the standard error.

	*η* (dB)		${\hat{\sigma }}_{\text{int}}$ (dB)		RMSe (dB)	
	0.5 c/deg	6 c/deg	0.5 c/deg	6 c/deg	0.5 c/deg	6 c/deg
AB	0.4 ± 0.5	0.1 ± 0.7	-0.3 ± 0.9	10.7 ± 1.2	0.33	0.71
AR	-0.2 ± 0.9	-4.7 ± 1.9	1.2 ± 1.8	17.6 ± 3.5	0.68	1.99
DB	-0.5 ± 0.6	-0.9 ± 1.0	1.3 ± 0.8	16.5 ± 1.5	0.74	0.18
GS	-1.6 ± 0.8	-2.2 ± 0.6	2.3 ± 1.0	13.5 ± 1.0	1.41	1.26
JZ	-0.5 ± 0.5	-0.9 ± 0.7	2.2 ± 0.9	10.2 ± 1.3	0.90	0.87
Mean	-0.5 ± 0.3	-1.7 ± 0.8	1.4 ± 0.5	13.7 ± 1.5		

It is expected that the NLM curves will have a very similar shape to those from the LAM (aside from a small facilitation at very low noise levels due to stochastic resonance [35]). We are interested in how changes in the parameters of the NLM will be reflected in the LAM fit, therefore it is not necessary to collect data at more mask levels than would ordinarily be used to characterise the LAM. We fit the simulated NLM data with the LAM in order to demonstrate the fitted LAM parameters expected on the basis of the dipper functions we collected. These parameters are provided in Table 4, and the curves are shown in Fig 6. Note that although the main difference seen between the two spatial frequencies in Table 4 is the internal noise ${\hat{\sigma }}_{\text{int}}$ of the fitted LAM function, most of this effect is actually arising as a result of the different *z* parameters in the nonlinear contrast response function (the *σ*_int_ used in our simulations varied only slightly between the spatial frequencies, based on the pedestal masking results). This is the reason why the fitted LAM ${\hat{\sigma }}_{\text{int}}$ is distinguished from the actual *σ*_int_ by decoration with a hat and illustrates one of the key points of this paper: changes in the fitted LAM ${\hat{\sigma }}_{\text{int}}$ may not (and *do not* in this case) correspond to changes in the actual *σ*_int_ in a nonlinear system.

**Table 4:** Parameters (in dB) obtained by fitting the LAM model to simulated nonlinear model (NLM) data, that were generated based on each observer’s dipper function. Standard errors provided are calculated from the bootstrap distributions. Predictions for these parameters are shown by the solid curves in Fig 6. The mean across observers is reported with the standard error.

	*η* (dB)		${\hat{\sigma }}_{\text{int}}$ (dB)	
	0.5 c/deg	6 c/deg	0.5 c/deg	6 c/deg
AB	-0.17 ± 0.29	-0.04 ± 0.32	1.0 ± 0.5	14.5 ± 0.5
AR	-0.81 ± 0.28	0.29 ± 0.42	-0.5 ± 0.7	28.2 ± 0.7
DB	-0.24 ± 0.29	0.15 ± 0.34	1.8 ± 0.5	18.7 ± 0.5
GS	-0.63 ± 0.29	0.29 ± 0.34	4.1 ± 0.6	17.7 ± 0.5
JZ	-0.23 ± 0.28	0.20 ± 0.31	2.2 ± 0.5	13.9 ± 0.6
Mean	-0.42 ± 0.13	0.18 ± 0.06	1.7 ± 0.8	18.6 ± 2.6

The NLM prediction curves in Fig 6 are generally close to those from the LAM fits. At 0.5 c/deg the curves are very similar for all observers. At 6 c/deg there is some disagreement, however for all observers except AR it is only at the left hand side where the internal noise is dominant. The predictions then converge at the high mask levels. This indicates a small difference in the internal noise (${\hat{\sigma }}_{\text{int}}$), but very similar efficiency. In Table 3 we see that the fitted efficiency *η* is close to 0 dB (100%) in all cases apart from the 6 c/deg condition for AR. The efficiency values in Table 4 are also close to 0 dB, though there is no component of the nonlinear model simulating these data that behaves like the efficiency parameter in the LAM. The deviations from 0 dB are simply due to the stochastic nature of the nonlinear model. A parameter controlling efficiency could be built into this model (see Appendix B), however we did not include it because our noise masking results indicate that efficiency is close to 100% for this task [44, 72]. Note that we report efficiency as

$$\begin{array}{c} 20\times \log_{10}(\eta ), \end{array}$$ (14)

here, which is equivalent to the use of

$$\begin{array}{c} 10\times \log_{10}{\left(\frac{\text{observed}\;d^{\prime }}{\text{ideal}\;d^{\prime }}\right)}^{2}, \end{array}$$ (15)

by some previous studies. By comparing the internal noise parameters between Tables 3 and 4 we can see that at 0.5 c/deg they are very similar, however at 6 c/deg the ${\hat{\sigma }}_{\text{int}}$ obtained by fitting to the data is slightly lower than that expected based on the nonlinear simulation. We will return to this in our bootstrap analysis below. Note that the reason why the standard errors in Table 4 are so much smaller than those in Table 3 is that the underlying dataset used to generate Table 4 is from the pedestal masking experiment, whereas Table 3 is generated based on the noise masking experiment data.

**Fig 7:**
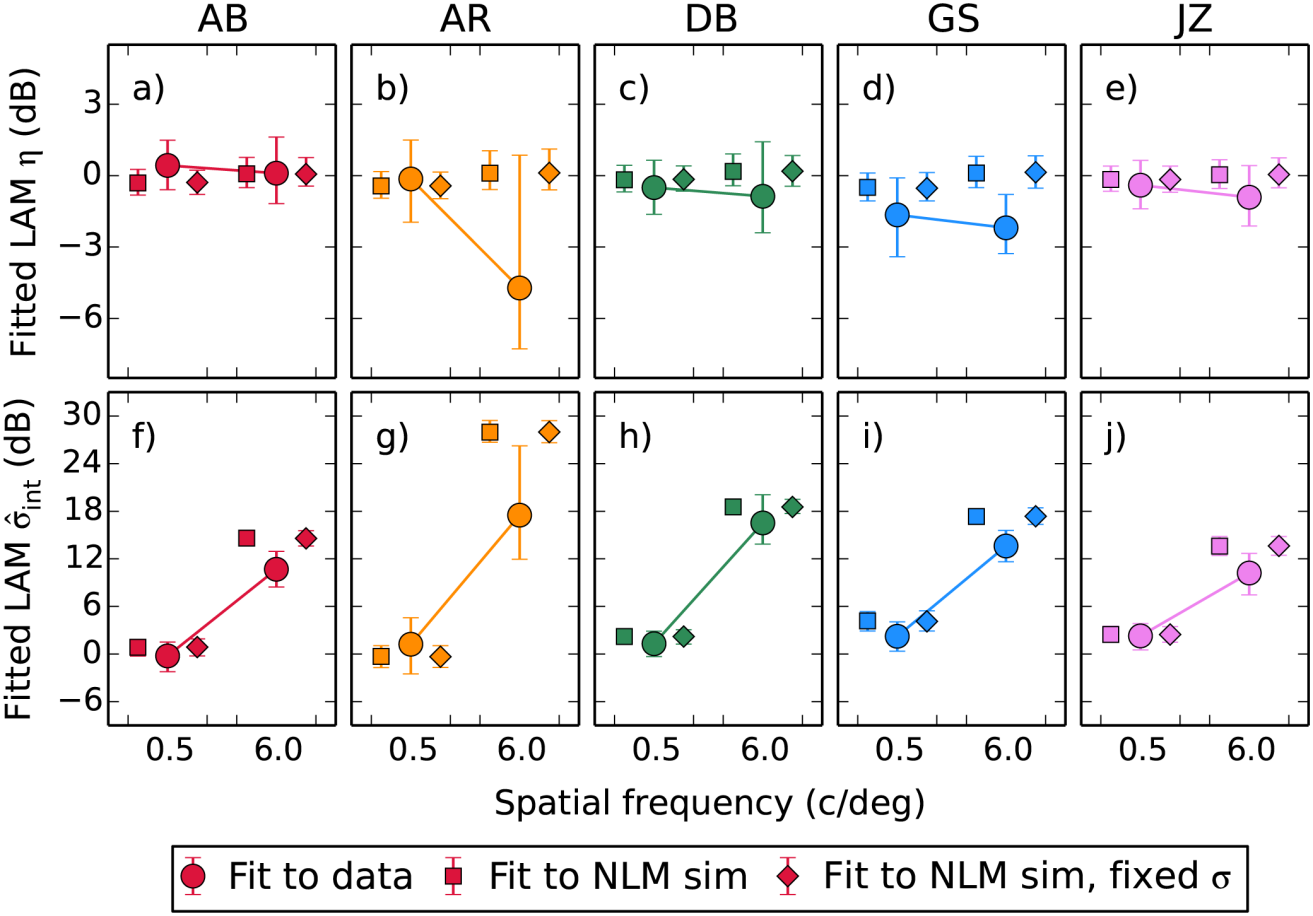
Parameters from LAM fits to simulated data from the nonlinear model (using the parameter populations presented in Fig 5) compared against the parameters (medians with 95% confidence intervals) obtained by fitting the bootstrapped empirical thresholds.

We conducted a further analysis based on bootstrapping in order to get an idea of the size of our effects relative to the variability in the data. The thousand sets of thresholds generated during the bootstrapping process were each fitted with the LAM model, allowing us to plot the median value of each parameter here with 95% confidence intervals. We were also able to generate a bootstrapped version of the nonlinear model prediction using the parameters summarised in Fig 5 (where the bootstrapping was performed on the dipper function data to generate a population of CRF parameters). At this point we also introduce another version of the nonlinear model, that where the internal noise *σ*_int_ parameter used in the simulation process is fixed across the two spatial frequencies (set at the mean of two fitted *σ*_int_ values). This allows us to demonstrate that the large differences in the fitted ${\hat{\sigma }}_{\text{int}}$ value in the noise masking experiment are due to differences in *z*. This comparison can be made in Fig 7: predictions from the nonlinear model with variable *σ*_int_ are shown by square symbols, and the fixed *σ*_int_ by diamonds. There is little difference between them.

Comparing the nonlinear simulations in Fig 7 against the parameters obtained by fitting to the bootstrapped data, the behaviour at 0.5 c/deg is predicted very accurately by our model. At 6 c/deg the median parameter estimates from AR indicate lower efficiency and internal noise than we predict, however there is a large variability associated with these. The 95% confidence intervals for the efficiency parameter overlap with our prediction, and those for the internal noise parameter approach it. For other observers there is a tendency for the fitted ${\hat{\sigma }}_{\text{int}}$ to be slightly lower at 6 c/deg than we predict. This residual effect may be due to a task-dependent change in either the internal noise *σ*_int_ or the saturation constant *z*. In general however we can account for most of the change in ${\hat{\sigma }}_{\text{int}}$ with a generating model based on the measured changes in *z* with a fixed *σ*_int_, indicating that in this case the result seen in the “internal noise” parameter of the noise masking data is not due to noise.

## Discussion

### Noise masking confounds internal noise measurements with nonlinearities

Our results demonstrate that effects which appear to be changes in internal noise when measured by the noise masking paradigm may instead be due to changes in gain control properties. Specifically, an increased saturation constant *z* behaves like a change in internal noise. Other studies [33] have previously suggested a noise masking model based on contrast gain control effects, and a recent meta-analysis [34] showed that *z* was more likely responsible for individual differences in contrast sensitivity than *σ*_int_. The work we present here however is the first time that the relationship between the two paradigms has been directly explored, thanks to the use of the “zero-dimensional” contrast-jitter noise paradigm. This allows us to present the same stimuli for our noise and pedestal masking experiments. We show that one can use the contrast response function obtained from a pedestal masking experiment to mostly predict an observer’s behaviour in a noise masking experiment, and that when doing so the human decision stage approaches ideal performance. Demonstrating this result with the more conventional white- or pink-noise masking method would also require a model of how the off-target stimulation from the broadband noise mask serves to suppress the detection of the target [43]. To our knowledge however these effects are typically not considered, and there is no noise masking model that takes account of them.

### Internal noise does not change much with spatial frequency

Although the primary purpose of this paper was as a proof-of-concept, with the manipulation of spatial frequency chosen as an example, our results still provide a novel insight into why sensitivity declines with spatial frequency. We must first estimate how much of the sensitivity loss can be attributed to optical factors. The lowpass Optical Transfer Function (OTF) of the eye attenuates higher spatial frequencies more than lower spatial frequencies. Attenuation by the eye’s optics will reduce the input gain of the system. In Eq (11) an input gain parameter *g* could be incorporated

$$\begin{array}{c} \text{f}\left(c\right)=\frac{{\left(gc\right)}^{p}}{z+{\left(gc\right)}^{q}}, \end{array}$$ (16)

however when this is done the effects of the new parameter overlap almost entirely with those of the existing *z* parameter. To show this we set the *z* parameter in Eq (16) to 1 and then adjusted the *g* parameter to fit the output of Eq (11) with a varying *z* parameter. We also set the *p* and *q* parameters to 2.4 and 2, as was the case in our experiments. A log-log plot of the best-fitting *g* values against the values of *z* used in the generating model gives a straight line (within the range of *z* values we find in this experiment). The parameters from a straight line fit then allow us to say that for an *x* dB change in *z*, we expect a 0.41*x* dB translation both upward and to the right in the dipper functions. This can be confirmed by comparing the differences in the *z* values in Table 2 against the dipper functions in Fig 3.

To calculate our expected optical effects we will refer to the formula provided by Watson [73], who developed a descriptive model to account for the OTF of young healthy eyes. The shape of the OTF depends on the pupil diameter, with wider pupils resulting in a steeper decline. For the observers tested at McGill the diameters were within the range of 2.5-3.5 mm. The expected relative attenuation difference between our two spatial frequencies will be in the range of 2.0-3.0 dB for pupils of 2.5-3.5 mm. This is equivalent to a 4.9-7.3 dB change in *z*. For DB the pupil size was 5.5 mm. This would give an attenuation difference of 7.7 dB, equivalent to a 18.8 dB change in *z*. By comparing these values against the differences in *z* reported in Table 2 one can see that optical factors account for only a small part of the shift in the dipper functions for the observers tested at McGill. For observer DB the effect attributable to optical effects is larger (half of the change in *z*). One must take these effects into account, particularly in situations where comparisons are made between groups of subjects in whom differences in OTF may be expected (e.g. ageing populations [74]).

Once the optical effects are accounted for the remaining difference in *z* should have a neural basis. One possible explanation is that performance at different spatial frequencies is limited by the number of cone photoreceptors contributing to the receptive field [75, 76]. An ideal combination of cone outputs within the footprint of a receptive field predicts large differences in threshold between our two spatial frequencies (>18 dB in the appendix of Baldwin, Meese & Baker’s study [54] which is far bigger than the neural effect left to be explained). However, if sensitivity is limited by cone density alone then the predicted threshold vs. eccentricity functions for different spatial frequency stimuli are parallel when eccentricity is expressed in degrees, whereas in reality they are parallel when eccentricity is expressed relative to stimulus wavelength [54, 77]. The steepening of the OTF with eccentricity would partially correct the cone-sampling prediction *toward* scale invariance, but the magnitude predicted for that effect from previous data is far too small to actually reach it [78]. It is possible to envision different (non-ideal) sampling strategies, or weights on the gain applied to the tuned outputs, that would result in a scale invariant system with a neural transfer function matching our results. Such a model could not be constrained by our data. A third possible neural factor would be divisive suppression from contrast gain control processes, which is what the *z* parameter in our contrast response function can be considered to represent.

The results we report here are the effects seen over only a relatively small distance in the spatial and temporal frequency space. It is possible that comparisons between more remote spatial frequencies or between different temporal frequencies would demonstrate greater changes in internal noise. Another possibility is that there is a single limiting source of noise for all contrast detection tasks, in which case all differences in contrast sensitivity would be attributed to differences in gain.

### Implications for the noise masking literature

Our key finding is that one cannot report changes in the fitted internal noise parameter of a LAM function as actual changes in internal noise without first establishing that the system being investigated is linear. We also show that the noise masking paradigm itself confounds changes in internal noise with changes in the nonlinearity, meaning that an alternative model that did feature those effects could not be constrained by noise masking data alone. Examples of these nonlinearity effects would be changes in sampling of the input, the gain, or the subsequent divisive inhibition within a contrast gain control process. Where previous noise masking experiments have found effects attributed to internal noise these may in fact be partially or wholly due to changes in these nonlinear aspects of the system being investigated. While it is true that some of these studies were carried out with the knowledge that the effects they are attributing to “equivalent internal noise” differences may have multiple possible explanations (the majority of which being unrelated to actual changes in noise) these caveats are often left unexpressed. Where changes in the internal noise in a system are of interest one can use pedestal masking experiments to distinguish between those effects and those due to differences in the nonlinear response. The inadequacy of using the noise masking method on its own to constrain our interpretation of the actual processes occurring in the brain is only supported by Dao, Lu & Dosher [79], who in their appendices demonstrate how the PTM can produce equivalent noise masking results to a model based on contrast gain control. A more sophisticated experiment is necessary to distinguish between the two [44].

## Appendix A: Using maximum likelihood to fit the CRF

**Fig 8:**
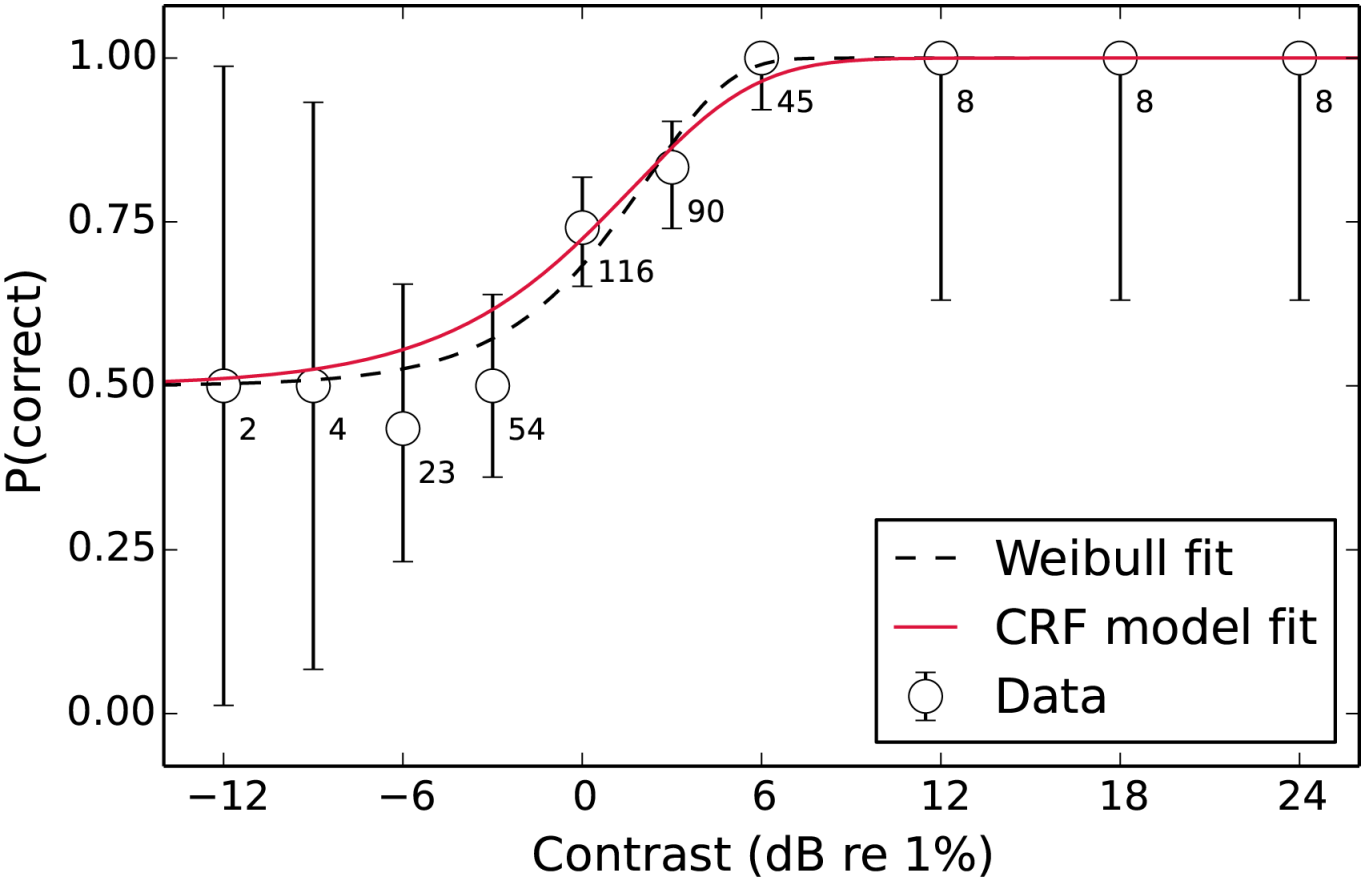
Example psychometric function (unmasked detection threshold for observer DB with binomial error bars) showing the fits from both the Weibull psychometric function and the CRF model. Each data point is labelled with the number of trials tested at that contrast level. The Weibull fit is made just to the data presented in this figure, whereas the CRF fit is made to the data from all mask levels simultaneously.

The typical method [28] used to find the CRF is by performing an experiment where the threshold target contrast (*t*_thresh_) corresponding to a particular *d*′ (e.g. *d*′ ≈ 1.273 if *t*_thresh_ is the *α* parameter of a fitted Weibull function) is found for multiple mask levels (the vector ***m***). An iterative search procedure is then used to find the values of the CRF parameters (*p*, *q*, *z* & *σ*_int_) that minimise the difference between the model’s predicted set of *t*_thresh_ values and those found in the data. This method has several disadvantages: i) the initial psychometric function fitting stage that finds the empirical *t*_thresh_ values requires sufficient data to perform a stable fit at each mask level in ***m***, ii) because only the threshold parameter of the psychometric function is used, the information from the slope of the psychometric function is discarded, and iii) the nested search that is used to find the predicted *t*_thresh_ for each set of parameters slows down the fitting procedure. All these disadvantages can be avoided using a maximum-likelihood fitting procedure [65, 81, 82]. This method takes advantage of the fact that each set of parameters put into Eq (11) describes a family of psychometric functions (one for each mask level, e.g. Fig 8). This entire family of functions can be fit simultaneously to the raw data (the number of trials ***n*** and correct responses ***c***) for each target level (***t***) and mask level (***m***). Using a maximum-likelihood method to choose the best-fitting parameters also allows the data to be weighted by the number of observations made for each condition. In this method, Eq (11) is used to find the *d*′ predicted by the current parameter set for the tested ***m*** and ***t***. These *d*′ values are then converted using the normal integral (Φ) to their corresponding percent-correct values for each condition (*i*)

$$\begin{array}{c} P_{i}\left(c\right)=\Phi (d_{i}^{\prime }), \end{array}$$ (17)

(for 2IFC the equation given for this step usually divides *d*′ by $\sqrt{2}$, but this is already accounted for in Eq (11) because *σ*_int_ represents the combined noise from both intervals). The log-likelihood of that parameter set is then calculated as

$$\begin{array}{c} \log L\left(p,q,z,\sigma_{\text{int}}|m,t\right)=\sum c_{i}\log[P_{i}\left(c\right)]+\left(n_{i}-c_{i}\right)\log[1-P_{i}\left(c\right)]. \end{array}$$ (18)

This is derived from the likelihood function

$$\begin{array}{c} L\left(p,q,z,\sigma_{\text{int}}|m,t,n,c\right)=\prod L_{i}, \end{array}$$ (19)

where the combined likelihood L is the product of the likelihoods for each condition

$$\begin{array}{c} L_{i}=L\left(p,q,z,\sigma_{\text{int}}|m_{i},t_{i},n_{i},c_{i}\right). \end{array}$$ (20)

These likelihoods are based on the probability that each of the trial-by-trial responses (correct and incorrect) made in that condition would occur, given the current parameter set. They are calculated using the “and rule”, where *P*(*A* ∩ *B*) = *P*(*A*)*P*(*B*)

$$\begin{array}{c} L_{i}=\prod_{j=1}^{c_{i}}P(c|m_{i},t_{i};p,q,z,\sigma_{\text{int}})\prod_{k=1}^{n_{i}-c_{i}}[1-P(c|m_{i},t_{i};p,q,z,\sigma_{\text{int}})]. \end{array}$$ (21)

Taking *P*_*i*_(*c*) = *P*(*c*|*m*_*i*_, *t*_*i*_;*p*, *q*, *z*, *σ*_int_), we can simplify this (because the values of *j* and *k* are not used) and rewrite Eq (19) as

$$\begin{array}{c} L\left(p,q,z,\sigma_{\text{int}}|m,t,n,c\right)=\prod P_{i}{\left(c\right)}^{c_{i}}{\left[1-P_{i}\left(c\right)\right]}^{n_{i}-c_{i}}. \end{array}$$ (22)

Taking the log of Eq (22) gives us Eq (18). Fitting the log-likelihood function lets us avoid using very small numbers that may fall below the precision limit of the software used for the computation. It also makes the calculation faster due to the nature of the mathematical operations involved.

The best-fitting parameters are found by using the fminsearch function in Matlab to minimise the negative log-likelihood (-logL). This is equivalent to finding the maximum likelihood solution.

## Appendix B: What role is left for efficiency?

In our experiments we find efficiency to be close to that of an ideal observer. Previous noise masking studies have typically found lower efficiencies. One reason for this difference will be the type of masking noise used. A broadband noise mask will likely cause uncertainty about the expected target, and so reduce efficiency [83–85]. Another possible explanation involves spatial summation: if a target is detected by multiple mechanisms (e.g. spatially adjacent receptive fields) then they must be able to linearly pool their outputs together in order to achieve ideal performance. If the combination is nonlinear then performance will fall below the ideal [86].

**Fig 9:**
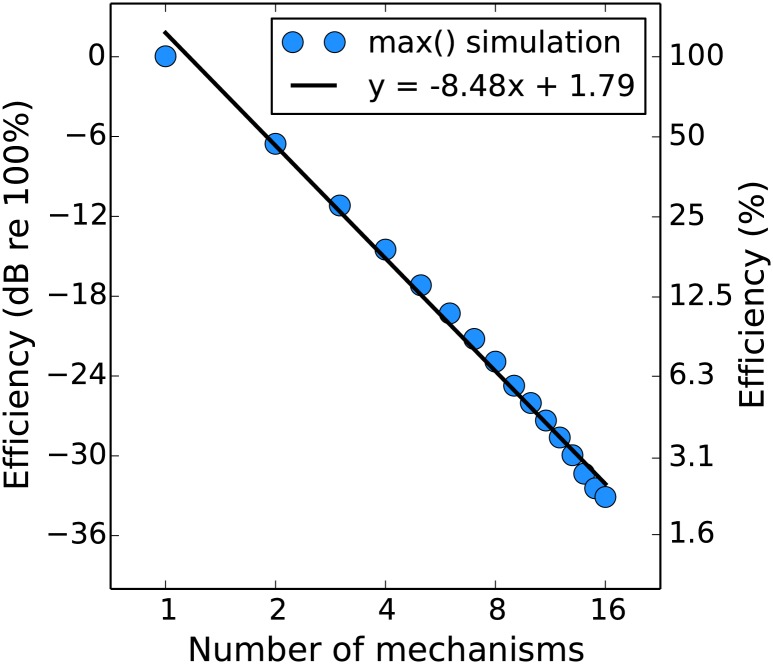
Efficiency of an observer who combines signals over multiple channels by picking the maximum, expressed relative to that of a linear ideal observer. We obtained by simulating the detection of various levels of signal (-42 to 36 dB in 3 dB steps) by independently noisy channels with different standard deviations (6 to 18 dB in 3 dB steps). We simulated 5,000 trials per combination of signal and noise level, both for a system where the outputs were summed (ideal) before comparison between the two intervals and for a system where the max() was taken. The data from this simulation were fit by a psychometric function (see Methods), and then the average efficiency of the max() observer was calculated relative to the ideal.

The greatest nonlinearity is a max() operator, where only the response of the mechanism which is most activated by the stimulus in each interval contributes to the decision as to which interval contained the target. We used the stochastic Monte Carlo method to generate efficiency predictions for a system that behaves in this way (with *n* spatially-adjacent contrast-detecting mechanisms that are affected by uncorrelated noise), relative to that of an ideal observer. This is shown in Fig 9 (see caption for simulation details). As the number of relevant mechanisms increases the efficiency drops dramatically. The max() operator can be considered as Minkowski summation with an infinitely high exponent (*m* = ∞)

$$\begin{array}{c} r_{\text{output}}={\left(\sum_{i=1}^{n}r_{i}^{m}\right)}^{\frac{1}{m}}, \end{array}$$ (23)

therefore as the exponent decreases we expect efficiency to increase (as the other mechanisms which are not the most activated begin to make more of a contribution) from that shown in Fig 9 and eventually reach ideal performance (*y* = 0) when the exponent is 1.

This raises the question of why our results do not show overall lower efficiencies, considering that we find nonlinear signal transduction in our pedestal masking experiment. Our explanation is that we used a small target which was approximately the size of a V1 receptive field. In previous summation experiments [53, 87] it has been demonstrated that spatial summation over such a short range (considered to be “within” a V1 receptive field) is linear, with nonlinear summation occurring for larger stimuli. It is possible that if we increased the size of our stimuli we would see a decline in efficiency (as has been shown previously in broadband noise [86]). If this effect were sufficiently large it could be used to measure the receptive field size of the linear mechanisms. A similar situation in which we would expect to see an effect on efficiency is if the observer is operating in conditions of uncertainty. Note that the work in this appendix is related to how Birdsall’s Theorem breaks down in multichannel systems [88, 89].

## References

[1] Faisal AA, Selen LPJ, Wolpert DM. Noise in the nervous system. Nature Reviews Neuroscience. 2008;9:292–303. 10.1038/nrn2258 18319728 PMC2631351

[2] Green DM, Swets JA. Signal Detection Theory and Psychophysics. 1988th ed Los Altos, California: Peninsula Publishing; 1966.

[3] Gregory RL, Cane V. A statistical information theory of visual thresholds. Nature. 1955;176:1272–1272. 10.1038/1761272a0 13321882

[4] Swets JA. Is there a sensory threshold? Science. 1961;134(3473):168–177. 10.1126/science.134.3473.168 13774294

[5] Corso JF. A theoretico-historical review of the threshold concept. Psychological Bulletin. 1963;60(4):356–370. 10.1037/h0040633 14041608

[6] Rose A. The sensitivity performance of the human eye on an absolute scale. Journal of the Optical Society of America. 1948 2;38(2):196–208. 10.1364/JOSA.38.000196 18901781

[7] Barlow HB. Retinal noise and absolute threshold. Journal of the Optical Society of America. 1956 8;46(8):634–639. 10.1364/JOSA.46.000634 13346424

[8] Nagaraja NS. Effect of luminance noise on contrast thresholds. Journal of the Optical Society of America. 1964;54(7):950–955. 10.1364/JOSA.54.000950

[9] Legge GE, Kersten D, Burgess AE. Contrast discrimination in noise. Journal of the Optical Society of America A. 1987 2;4(2):391–404. 10.1364/JOSAA.4.000391 3559785

[10] Lu ZL, Dosher BA. Characterizing human perceptual inefficiencies with equivalent internal noise. Journal of the Optical Society of America A, Optics, image science, and vision. 1999 3;16(3):764–778. 10.1364/JOSAA.16.000764 10069062

[11] Pelli DG, Farell B. Why use noise? Journal of the Optical Society of America A. 1999 3;16(3):647–53. 10.1364/JOSAA.16.000647 10069051

[12] Neri P. The statistical distribution of noisy transmission in human sensors. Journal of Neural Engineering. 2013;10(016014):1–13. 10.1088/1741-2560/10/1/016014 23337440

[13] Pelli DG. Effects of Visual Noise. Cambridge University; 1981.

[14] McAnany JJ, Alexander KR. Spatial contrast sensitivity in dynamic and static additive luminance noise. Vision Research. 2010;50:1957–1965. 10.1016/j.visres.2010.07.006 20638404 PMC2926298

[15] Kersten D, Hess R, Plant GT. Assessing contrast sensitivity behind cloudy media. Clinical Vision Science. 1988;2(3):143–158.

[16] Pelli DG, Levi DM, Chung STL. Using visual noise to characterize amblyopic letter identification. Journal of Vision. 2004;4:904–920. 10.1167/4.10.6 15595894 PMC2751822

[17] McAnany JJ, Alexander KR, Genead MA, Fishman GA. Equivalent intrinsic noise, sampling efficiency, and contrast sensitivity in patients with retinitis pigmentosa. Investigative Ophthalmology and Visual Science. 2013;54(6):3857–3862. 10.1167/iovs.13-11789 23661376 PMC3671935

[18] Bennett PJ, Sekuler AB, Ozin L. Effects of aging on calculation efficiency and equivalent noise. Journal of the Optical Society of America A. 1999;16(3):654–668. 10.1364/JOSAA.16.000654 10069052

[19] Pardhan S. Contrast sensitivity loss with aging: sampling efficiency and equivalent noise at different spatial frequencies. Journal of the Optical Society of America A. 2004;21(2):169–175. 10.1364/JOSAA.21.000169 14763759

[20] Allard R, Renaud J, Molinatti S, Faubert J. Contrast sensitivity, healthy aging and noise. Vision Research. 2013;92:47–52. 10.1016/j.visres.2013.09.004 24070688

[21] Christensen BK, Spencer JMY, King JP, Sekuler AB, Bennett PJ. Noise as a mechanism of anomalous face processing among persons with Schizophrenia. Frontiers in Psychology. 2013;4(401):1–10. 23882228 10.3389/fpsyg.2013.00401 PMC3712139

[22] Legge GE, Foley JM. Contrast masking in human vision. Journal of the Optical Society of America. 1980;70(12):1458–1471. 10.1364/JOSA.70.001458 7463185

[23] Cannon MW, Fullenkamp SC. A transducer model for contrast perception. Vision Research. 1991;31(6):983–998. 10.1016/S0042-6989(05)80001-X 1858328

[24] Heeger DJ. Normalization of cell responses in cat striate cortex. Visual Neuroscience. 1992;9:181–197. 1504027 10.1017/s0952523800009640

[25] Boynton GM, Demb JB, Glover GH, Heeger DJ. Neuronal basis of contrast discrimination. Vision Research. 1999 1;39:257–269. 10.1016/S0042-6989(98)00113-8 10326134

[26] Baker DH, Vilidaite G. Broadband noise masks suppress neural responses to narrowband stimuli. Frontiers in Psychology. 2014;5(763):1–9. 25076930 10.3389/fpsyg.2014.00763 PMC4098025

[27] Foley JM. Human luminance pattern-vision mechanisms: masking experiments require a new model. Journal of the Optical Society of America A. 1994;11(6):1710–1719. 10.1364/JOSAA.11.001710 8046537

[28] Meese TS, Georgeson MA, Baker DH. Binocular contrast vision at and above threshold. Journal of Vision. 2006;6(11):1224–43. 10.1167/6.11.7 17209731

[29] Meese TS, Summers RJ. Area summation in human vision at and above detection threshold. Proceedings of the Royal Society B. 2007;274:2891–2900. 10.1098/rspb.2007.0957 17851151 PMC2211515

[30] Goris RLT, Putzeys T, Wagemans J, Wichmann FA. A neural population model for visual pattern detection. Psychological Review. 2013;120(3):472–496. 10.1037/a0033136 23915083

[31] Busse L, Wade AR, Carandini M. Representation of concurrent stimuli by population activity in visual cortex. Neuron. 2009;64:931–942. 10.1016/j.neuron.2009.11.004 20064398 PMC2807406

[32] Tolhurst DJ, Heeger DJ. Comparison of contrast-normalization and threshold models of the responses of simple cells in cat striate cortex. Visual Neuroscience. 1997;14:293–309. 10.1017/S0952523800011433 9147482

[33] Hess RF, Baker DH, May KA, Wang J. On the decline of 1st and 2nd order sensitivity with eccentricity. Journal of Vision. 2008;8:1(19):1–12. 10.1167/8.1.19 18318622

[34] Baker DH. What is the primary cause of individual differences in contrast sensitivity? PloS One. 2013 1;8(7):1–9. 10.1371/journal.pone.0069536 PMC3724920 23922732

[35] Goris RLT, Zaenen P, Wagemans J. Some observations on contrast detection in noise. Journal of Vision. 2008;8:9(4):1–15. 10.1167/8.9.4 18831640

[36] Baldwin AS, Meese TS. Fourth-root summation of contrast over area: no end in sight when spatially inhomogeneous sensitivity is compensated by a witch’s hat. Journal of Vision. 2015;15:15(4):1–12. 10.1167/15.15.4 26575190

[37] Baker DH, Meese TS. Contrast integration over area is extensive: a three-stage model of spatial summation. Journal of Vision. 2011;11(14)(14):1–16. 10.1167/11.14.14 22178702

[38] Meese TS, Summers RJ. Theory and data for area summation of contrast with and without uncertainty: evidence for a noisy energy model. Journal of Vision. 2012;12(11)(9):1–28. 10.1167/12.11.9 23077206

[39] Xu P, Lu ZL, Qiu Z, Zhou Y. Identify mechanisms of amblyopia in Gabor orientation identification with external noise. Vision Research. 2006;46:3748–3760. 10.1016/j.visres.2006.06.013 16904719

[40] Levi DM, Klein SA. What limits performance in the amblyopic visual system: Seeing signals in noise with an amblyopic brain. Journal of Vision. 2008;8:4(1):1–23. 10.1167/8.4.1 18484840

[41] Lu ZL, Dosher BA. Characterizing observers using external noise and observer models: assessing internal representations with external noise. Psychological Review. 2008;115(1):44–82. 10.1037/0033-295X.115.1.44 18211184

[42] Klein SA, Levi DM. Stochastic model for detection of signals in noise. Journal of the Optical Society of America A. 2009 11;26(11):B110–B126. 10.1364/JOSAA.26.00B110 PMC2942087 19884912

[43] Baker DH, Meese TS. Zero-dimensional noise: the best mask you never saw. Journal of Vision. 2012;12:10(20):1–12. 10.1167/12.10.20 23024357

[44] Baker DH, Meese TS. Regarding the benefit of zero-dimensional noise. Journal of Vision. 2013;13:10(26):1–6. 10.1167/13.10.26 23995501

[45] Gorea A, Sagi D. Disentangling signal from noise in visual contrast discrimination. Nature Neuroscience. 2001 11;4(11):1146–1150. 10.1038/nn741 11687818

[46] Brouwer GJ, Heeger DJ. Cross-orientation suppression in human visual cortex. Journal of Neurophysiology. 2011;106:2108–2119. 10.1152/jn.00540.2011 21775720 PMC3214101

[47] Chen G, Hou F, Yan FF, Zhang P, Xi J, Zhou Y, et al Noise provides new insights on contrast sensitivity function. PloS One. 2014;9(3):1–10. 10.1371/journal.pone.0090579 PMC3953123 24626135

[48] Hou F, Lu ZL, Huang CB. The external noise normalized gain profile of spatial vision. Journal of Vision. 2014;14:13(9):1–14. 10.1167/14.13.9 PMC4528485 25391301

[49] Cohn TE. Detectability of a luminance increment: effect of superimposed random luminance fluctuation. Journal of the Optical Society of America A. 1976;66(12):1426–1428.

[50] Dakin SC. Information limit on the spatial integration of local orientation signals. Journal of the Optical Society of America A. 2001;18(5):1016–1026. 10.1364/JOSAA.18.001016 11336204

[51] Campbell FW, Green DG. Optical and retinal factors affecting visual resolution. Journal of Physiology. 1965;181:576–593. 10.1113/jphysiol.1965.sp007784 5880378 PMC1357668

[52] Speranza F, Moraglia G, Schneider BA. Binocular detection of masked patterns in young and old observers. Psychology and Aging. 2001;16(2):281–292. 10.1037/0882-7974.16.2.281 11405316

[53] Meese TS. Spatially extensive summation of contrast energy is revealed by contrast detection of micro-pattern textures. Journal of Vision. 2010;10(8)(14):1–21. 10.1167/10.8.14 20884589

[54] Baldwin AS, Meese TS, Baker DH. The attenuation surface for contrast sensitivity has the form of a witch’s hat within the central visual field. Journal of Vision. 2012;12(11)(23):1–17. 10.1167/12.11.23 23104816

[55] De Valois RL, Albrecht DG, Thorell LG. Spatial frequency selectivity of cells in macaque visual cortex. Vision Research. 1982;22:545–559. 10.1016/0042-6989(82)90113-4 7112954

[56] De Valois RL, De Valois KK. Striate Cortex In: Spatial Vision. Oxford: Oxford University Press; 1990 p. 94–146.

[57] García-Pérez MA. Forced-choice staircases with fixed step sizes: asymptotic and small-sample properties. Vision Research. 1998;38:1861–1881. 10.1016/S0042-6989(97)00340-4 9797963

[58] Prins N, Kingdom FAA. Palamedes: Matlab routines for analyzing psychophysical data. wwwpalamedestoolboxorg. 2009;(accessed 07/01/13).

[59] Tukey JW. Schematic plots In: Exploratory Data Analysis. limited pr ed. Addison-Wesley; 1970.

[60] Hoaglin DC, Mosteller F, Tukey JW. Boxplots and Batch Comparison In: Understanding Robust and Exploratory Data Analysis. New York: John Wiley & Sons; 1983.

[61] Bird CM, Henning GB, Wichmann FA. Contrast discrimination with sinusoidal gratings of different spatial frequency. Journal of the Optical Society of America A. 2002 7;19(7):1267–1273. 10.1364/JOSAA.19.001267 12095194

[62] Burton GJ. Contrast discrimination by the human visual system. Biological Cybernetics. 1981;40(1):27–38. 10.1007/BF00326678 7236750

[63] R Core Team. R: A Language and Environment for Statistical Computing. Vienna, Australia: R Foundation for Statistical Computing; 2014.

[64] May KA, Solomon JA. Four theorems on the psychometric function. PLoS One. 2013 1;8(10):1–34. 10.1371/journal.pone.0074815 PMC3790801 24124456

[65] Georgeson MA, Meese TS. Contrast discrimination and pattern masking: contrast gain control with fixed additive noise. Perception. 2004;34(Suppl):754–755.

[66] Meese TS, Summers RJ. Neuronal convergence in early contrast vision: binocular summation is followed by response nonlinearity and area summation. Journal of Vision. 2009;9(4)(7):1–16. 10.1167/9.4.7 PMC2807356 19757916

[67] Meese TS, Baker DH. A common rule for integration and suppression of luminance contrast across eyes, space, time, and pattern. i-Perception. 2013 1;4(1):1–16. 10.1068/i0556 23799184 PMC3690412

[68] Kingdom FAA, Prins N. Goodness-of-Fit In: Psychophysics: A Practical Introduction. London: Academic Press: an imprint of Elsevier; 2010 p. 226–228.

[69] Akaike H. A New Look at the Statistical Model Identification. IEEE Transactions On Automatic Control. 1974;19(6):716–723. 10.1109/TAC.1974.1100705

[70] Burnham KP, Anderson DR. Information and Likelihood Theory: A Basis for Model Selection and Inference In: Model Selection and Multimodel Inference. 2nd ed New York: Springer-Verlag; 2002 p. 49–97.

[71] Lasley DJ, Cohn TE. Why discrimination may be better than detection. Vision Research. 1981;21:273–278. 10.1016/0042-6989(81)90121-8 7269304

[72] Allard R, Faubert J. Zero-dimensional noise is not suitable for characterizing processing properties of detection mechanisms. Journal of Vision. 2013;13(10)(25):1–3. 10.1167/13.10.25 23995500

[73] Watson AB. A formula for the mean human optical modulation transfer function as a function of pupil size. Journal of Vision. 2013;13:6(18):1–11. 10.1167/13.6.18 23729769

[74] Guirao A, González C, Redondo M, Geraghty E, Norrby S, Artal P. Average optical performance of the human eye as a function of age in a normal population. Investigative Ophthalmology & Visual Science. 1999;40(1):203–213. 9888445

[75] Geisler WS. Physical limits of acuity and hyperacuity. Journal of the Optical Society of America A. 1984;1(7):775–782. 10.1364/JOSAA.1.000775 6747742

[76] Banks MS, Geisler WS, Bennett PJ. The physical limits of grating visibility. Vision Research. 1987;27(11):1915–1924. 10.1016/0042-6989(87)90057-5 3447346

[77] Pointer JS, Hess RF. The contrast sensitivity gradient across the human visual field: with emphasis on the low spatial frequency range. Vision Research. 1989;29(9):1133–1151. 10.1016/0042-6989(89)90061-8 2617861

[78] Navarro R, Artal P, Williams DR. Modulation transfer of the human eye as a function of retinal eccentricity. Journal of the Optical Society of America A. 1993;10(2):201–212. 10.1364/JOSAA.10.000201 8478746

[79] Dao DY, Lu ZL, Dosher BA. Adaptation to sine-wave gratings selectively reduces the contrast gain of the adapted stimuli. Journal of Vision. 2006 1;6:739–759. 10.1167/6.7.6 16895456

[80] Baldwin AS, Hess RF. Investigating the shape of the contrast sensitivity function using white, bandpass, and contrast jitter noise. Journal of Vision. 2014;14(10):1421 10.1167/14.10.1421

[81] Morgan M, Chubb C, Solomon JA. A’dipper’ function for texture discrimination based on orientation variance. Journal of Vision. 2008;8(11)(9):1–8. 10.1167/8.11.9 PMC4135071 18831603

[82] Goris RLT, Wagemans J, Wichmann FA. Modelling contrast discrimination data suggest both the pedestal effect and stochastic resonance to be caused by the same mechanism. Journal of Vision. 2008;8(15)(17):1–21. 10.1167/8.15.17 19146300

[83] Birdsall TG. Detection of a signal specified exactly with a noisy stored reference signal. The Journal of the Acoustical Society of America. 1960;32(8):1038–1045. 10.1121/1.1908274

[84] Pelli DG. Uncertainty explains many aspects of visual contrast detection and discrimination. Journal of the Optical Society of America A. 1985 9;2(9):1508–1532. 10.1364/JOSAA.2.001508 4045584

[85] Burgess AE, Colborne B. Visual signal detection. IV. Observer inconsistency. Journal of the Optical Society of America A. 1988;5(4):617–627. 10.1364/JOSAA.5.000617 3404312

[86] Kersten D. Spatial summation in visual noise. Vision Research. 1984;24(12):1977–1990. 10.1016/0042-6989(84)90033-6 6534022

[87] Foley JM, Varadharajan S, Koh CC, Farias MCQ. Detection of Gabor patterns of different sizes, shapes, phases and eccentricities. Vision Research. 2007;47:85–107. 10.1016/j.visres.2006.09.005 17078992 PMC1994823

[88] Pelli DG. Noise in the Visual System May Be Early In: Landy M, Movshon JA, editors. Computational Models of Visual Processing. Cambridge: MIT Press; 1991 p. 147–152.

[89] Baldwin AS. Appendix A: Birdsall’s Theorem. In: Pattern Integration in the Normal and Abnormal Human Visual System. Aston University: PhD Thesis; 2013. p. 210–213.

